
JOURNAL INFORMATION
==============================
NLM Title Abbreviation: ChemistryOpen
Journal ID: ChemistryOpen
NLM Journal ID: 101594811
Journal ID: open

ChemistryOpen

EISSN: 2191-1363
Publisher: Wiley

ARTICLE INFORMATION
==============================
PMCID: PMC3892195
PMID: 24551562
DOI: 10.1002/open.201380513
Article version: 1
Subjects: News

Spotlights on our sister journals: ChemistryOpen 5-6/2013

Print publication date: 2013 Dec
Electronic publication date: 2013 Dec 19
Volume: 2
Issue: 5-6
First page: 172
Last page: 175
Copyright: Copyright © 2013 WILEY-VCH Verlag GmbH & Co. KGaA, Weinheim
Copyright year: 2013
License: Re-use of this article is permitted in accordance with the Creative Commons Deed, Attribution 2.5, which does not permit commercial exploitation.
License URL: https://creativecommons.org/licenses/by/2.5/

==============================
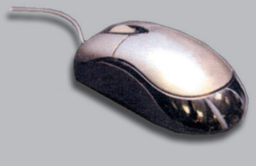

On these pages, we feature a selection of the excellent work that has recently been published in our sister journals. If you are reading these pages on a computer, click on any of the items to read the full article. Otherwise please see the DOIs for easy online access through Wiley Online Library.

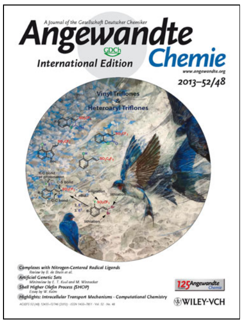

### Synthetic Erythropoiesis Protein

*S. B. H. Kent**

**Bringing the Science of Proteins into the Realm of Organic Chemistry: Total Chemical Synthesis of SEP (Synthetic Erythropoiesis Protein)**

**The glycoprotein mimetic** SEP (synthetic erythropoiesis protein) was prepared in homogenous form by total chemical synthesis. SEP had full biological activities and improved pharmacokinetic properties compared with first-generation recombinant human EPO. This work demonstrated that synthetic chemistry can be employed to systematically improve the properties of a protein biotherapeutic by the use of designed chemical moieties not found in nature.

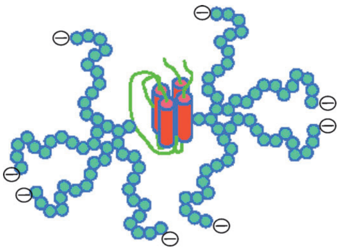

*Angew. Chem. Int. Ed.*
DOI: **10.1002/anie.201304116**

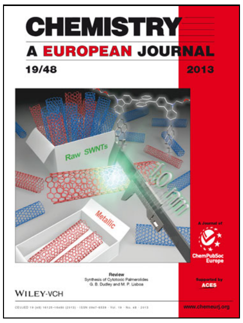

### Natural Products

*M. P. Lisboa, G. B. Dudley**

**Synthesis of Cytotoxic Palmerolides**

**Can synthesis deliver?** Chemical synthesis is the key to unlocking the chemotherapeutic potential of the palmerolides. This review covers the chemistry and biology of the palmerolides through early 2013, highlighting the evolution of a consensus strategy that could lead to cost-effective production of synthetic palmerolides. Palmerolide A is potently and selectively cytotoxic to certain melanomas. Its mechanism of action is poorly understood but likely involves inhibition of a transmembrane proton pump involved in regulation of cellular pH and metastasis.

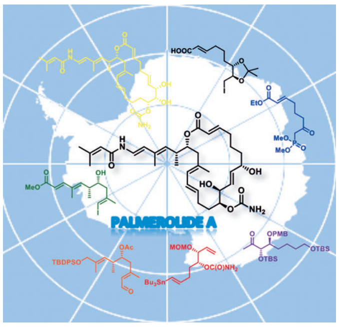

*Chem. Eur. J.*
DOI: **10.1002/chem.201302167**

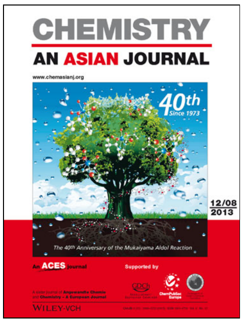

### Anode Materials

*H.-W. Shim, A.-H. Lim, J.-C. Kim, G.-H. Lee, D.-W. Kim**

**Hydrothermal Realization of a Hierarchical, Flowerlike $MnWO_4$@MWCNTs Nanocomposite with Enhanced Reversible Li Storage as a New Anode Material**

**Positively attracted**: A hierarchical, flowerlike $MnWO_4$-based composite with multiwalled carbon nanotubes (MWCNTs) was synthesized through a one-pot hydrothermal route and evaluated as new anodes for Li-ion batteries. Relative to bare $MnWO_4$ electrodes, the composite electrodes exhibit outstanding electrochemical behavior with increased cyclability and rate capability and a high specific capacity.

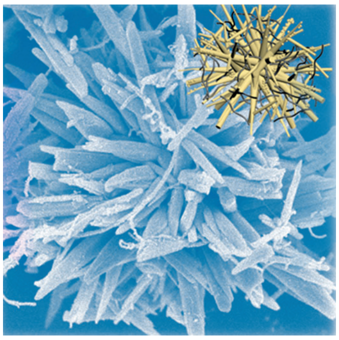

*Chem. Asian J.*
DOI: **10.1002/asia.201300765**

ChemPubSoc Europe

### TB Drug Screening

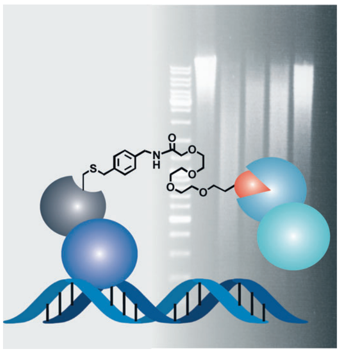

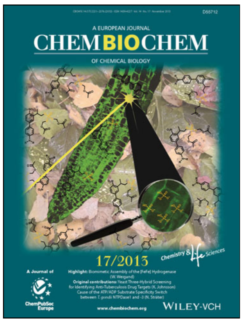

*S. Moser, K. Johnsson**

**Yeast Three-Hybrid Screening for Identifying Anti-Tuberculosis Drug Targets**

**Mycobacterium goes yeast**: Target deconvolution of anti-tuberculosis drugs can be a very challenging task. Here we report a yeast 3-hybrid system that allows promising small molecules to be screened for protein targets of a pathogen in nontoxic yeast cells. The system employs libraries of randomly fragmented bacterial DNA and offers a technically simple alternative approach for target identification.

*ChemBioChem*
DOI: **10.1002/cbic.201300472**

### Catalysts

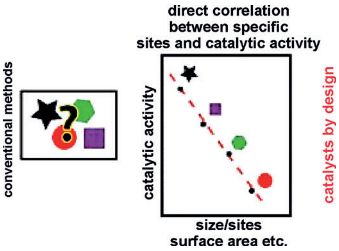

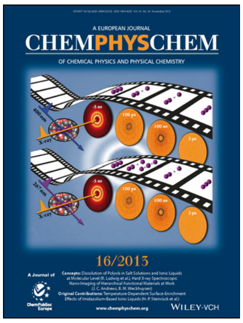

*M. Cargnello,* P. Fornasiero, R. J. Gorte*

**Playing with Structures at the Nanoscale: Designing Catalysts by Manipulation of Clusters and Nanocrystals as Building Blocks**

**Catalysts with improved activity,** stability and selectivity can be prepared by synthetic techniques that produce uniform building blocks, such as clusters and nanocrystals, and by appropriate manipulation and assembly at the nanoscale.

*ChemPhysChem*
DOI: **10.1002/cphc.201300702**

### Natural Product Chemistry

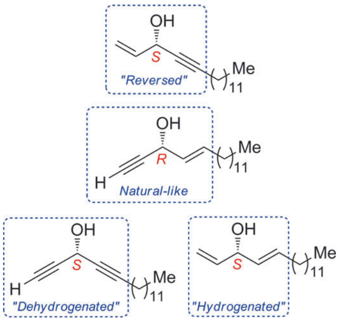

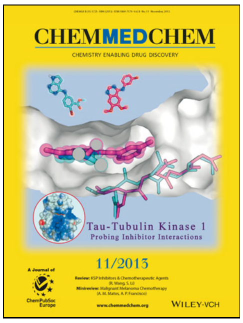

*D. El Arfaoui, D. Listunov, I. Fabing, M. Oukessou, C. Frongia, V. Lobjois, A. Samson, F. Ausseil, A. Ben-Tama, E. M. El Hadrami, R. Chauvin,* Y. Génisson**

**Identification of Chiral Alkenyl- and Alkynylcarbinols as Pharmacophores for Potent Cytotoxicity**

**Illumination by acetylene**: Systematic structural variations in a series of archetypal acetylenic lipids derived from the naturally occurring (*S*,*E*)-icos-4-en-1-yn-3-ol allowed the discovery of a series of 3*R*-like 1,4-di-unsaturated carbinol units with a significant and systematic enantiomeric effect on cytotoxicity.

*ChemMedChem*
DOI: **10.1002/cmdc.201300230**

### Hydrogen Production

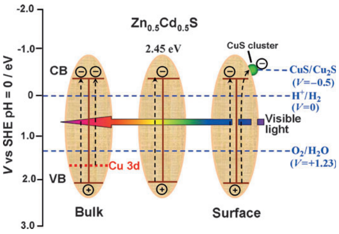

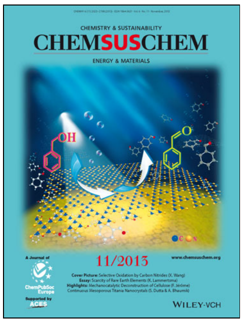

*J. Zhang, Q. Xu, S. Z. Qiao,* J. Yu**

**Enhanced Visible-Light Hydrogen-Production Activity of Copper-Modified $Zn_xCd_{1-x}S$**

**Bulked up!** Two different synthetic pathways are devised to prepare surface and bulk $Cu^{2+}$-modified $Zn_xCd_{1-x}S$ through cation-exchange and coprecipitation methods, respectively. $Cu^{2+}$ surface modification can provide a new electron-transferring pathway (interfacial charge transfer) and also form new surface active sites for $H_2$ evolution, resulting in enhanced visible-light $H_2$-production activity (see picture).

*ChemSusChem*
DOI: **10.1002/cssc.201300409**

© 2013 Wiley-VCH Verlag GmbH & Co. KGaA, Weinheim

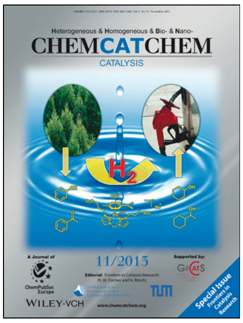

### Fischer-Tropsch

*B. Sun, J. Lin, K. Xu, Y. Pei, S. Yan, M. Qiao,* X. Zhang, B. Zong**

**Fischer–Tropsch Synthesis Over Skeletal Fe–Ce Catalysts Leached from Rapidly Quenched Ternary Fe–Ce–Al Alloys**

**$Ce^{III}$ works**: The promotion of rapidly quenched skeletal Fe with Ce significantly increases the selectivity to $C_{5+}$ hydrocarbons in the Fischer–Tropsch synthesis reaction, which is correlated with the surface concentration of Hägg carbide and interpreted as a consequence of the chemical promotion effect of Ce imparted by $Ce^{III}$. RQ = Rapidly quenched.

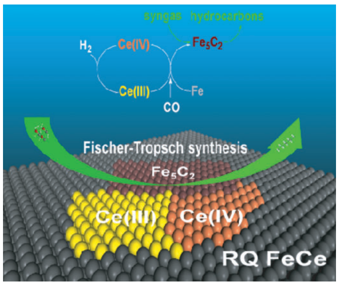

*ChemCatChem*
DOI: **10.1002/cctc.201300430**

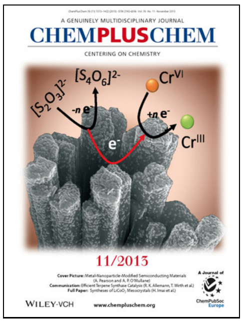

### High-Valent Iron

*O. Krahe, F. Neese,* M. Engeser**

**Iron Azides with Cyclam-Derived Ligands: Are They Precursors for High-Valent Iron Nitrides in the Gas Phase?**

**High-valent iron nitrides**: Azide complexes as their potential precursors are studied in the gas phase by mass spectrometry. A subtle interplay between pathways to $Fe^{II}$, $Fe^{III}$, and $Fe^{V}$ products is controlled by choice of the ligand (see figure). The observations partly parallel those seen after irradiating frozen solutions.

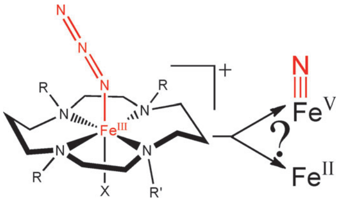

*ChemPlusChem*
DOI: **10.1002/cplu.201300182**

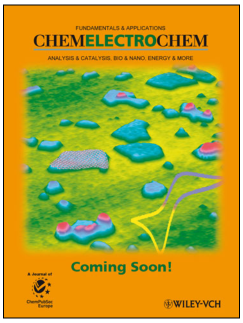

### Solar Cells

*J. Bisquert,* I. Mora-Sero, F. Fabregat-Santiago*

**Diffusion–Recombination Impedance Model for Solar Cells with Disorder and Nonlinear Recombination**

**Diffusing out**: The theory of diffusion–recombination impedance applied to nanostructured solar cells is presented with an emphasis on the effects of energy disorder and short diffusion length.

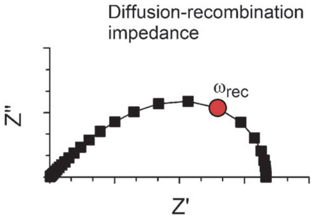

*ChemElectroChem*
DOI: **10.1002/celc.201300091**

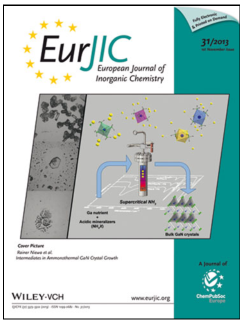

### HPA-Based Photocatalysts

*G. Marcì,* E. I. García-López, L. Palmisano*

**Heteropolyacid-Based Materials as Heterogeneous Photocatalysts**

The microreview describes the most significant contributions related to heteropoly acid (HPA) based materials used as heterogeneous photocatalysts. HPAs supported on different oxides (insulating or semiconductor) or associated to an inorganic, organic, or organometallic moiety are presented.

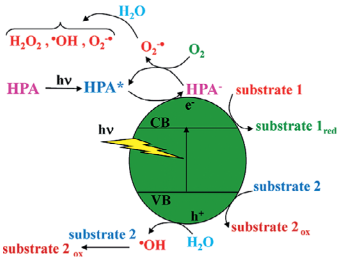

*Eur. J. Inorg. Chem.*
DOI: **10.1002/ejic.201300883**

© 2013 Wiley-VCH Verlag GmbH & Co. KGaA, Weinheim

### Ion-Pair Recognition

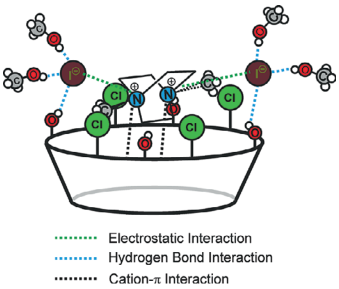

*Eur. J. Org. Chem.*
DOI: **10.1002/ejoc.201300886**

*N. Kodiah Beyeh, M. Göth, L. Kaufmann, C. A. Schalley,* K. Rissanen**

**The Synergetic Interplay of Weak Interactions in the Ion-Pair Recognition of Quaternary and Diquaternary Ammonium Salts by Halogenated Resorcinarenes**

The noncovalent interactions of upper-rim halogenated resorcinarenes with quaternary and diquaternary ammonium salts were analysed in the gas phase and in solution. Enhanced ion pair binding by a synergetic combination of cation···π and hydrogen-bond interactions was observed in solution, while the electronic effects of the substituents prevailed in the gas phase in the absence of the counteranion.

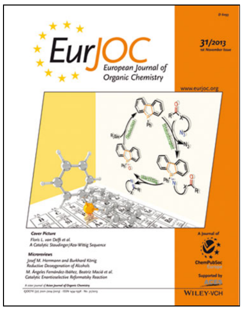

### Charge Transfer

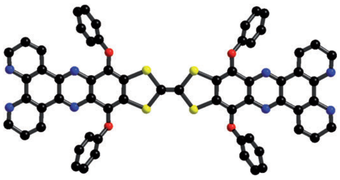

*Asian J. Org. Chem.*
DOI: **10.1002/ajoc.201300144**

*H. Jia, J. Ding, A. Hauser, S. Decurtins, S.-X. Liu**

**Large π-Conjugated Chromophores Derived from Tetrathiafulvalene**

**Make mine a large**: The direct annulation of two dipyrido[3,2-*a*:2′,3′-*c*]phenazine units to a tetrathiafulvalene core leads to a large π-conjugate as a bridging ligand. Its coordination ability to transition-metal ions is exemplified by the formation of a stable dinuclear $Ru^{2+}$ complex. The electrochemical, optical absorption, photoinduced intramolecular charge-transfer, and fluorescence characteristics of the ligand and the complex are described.

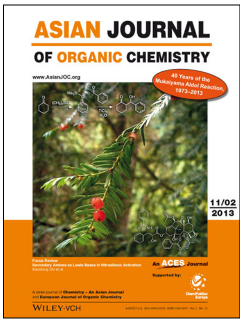

### Smart Materials

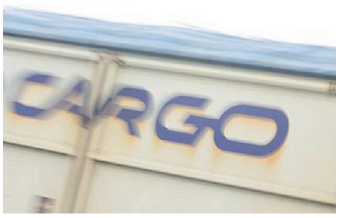

*ChemViews magazine*
DOI: **10.1002/chemv.201300113**

*David Bradley*

**Molecular Dockers Move Microscopic Cargoes**

Although the controlled movement and transportation of objects is easy to achieve at the macroscopic level, it is a much more complicated process at the microscopic level. This manipulation of microscopic particles was accomplished by Jérémie Palacci et al., USA, by using a cargo transport system based on self-propelled colloidal hematite particles, as David Bradley discusses.

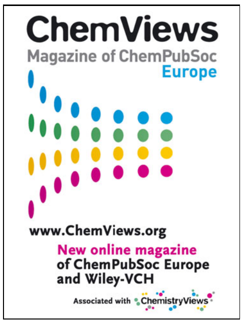

© 2013 Wiley-VCH Verlag GmbH & Co. KGaA, Weinheim
